# Acute Non‐Hemorrhagic Adrenal Infarction in a Diabetic Patient: A Rare but Critical Consideration in Acute Abdominal Pain

**DOI:** 10.1002/ccr3.70917

**Published:** 2025-10-14

**Authors:** Parvaneh Layegh, Atiyeh Mahdavi Rafie, Armin Doostparast

**Affiliations:** ^1^ Department of Radiology, Faculty of Medicine Mashhad University of Medical Sciences Mashhad Iran; ^2^ Student Research Committee, Faculty of Medicine Mashhad University of Medical Sciences Mashhad Iran

**Keywords:** acute abdominal pain, acute non‐hemorrhagic adrenal infarction, adrenal insufficiency, adrenal vein thrombosis, ANHAI

## Abstract

Acute non‐hemorrhagic adrenal infarction (ANHAI), though rare, should be taken into account in the differential diagnosis of patients presenting with unexplained abdominal pain, especially those with underlying hypercoagulable conditions or risk factors for thrombosis. Timely recognition and diagnosis are paramount to prevent complications, including adrenal insufficiency. Imaging plays a crucial role in diagnosing ANHAI, with CT scans typically showing an enlarged, hypo‐attenuated adrenal gland and the presence of the capsular sign offering valuable diagnostic clues. Here, we present a middle‐aged diabetic woman who presented with severe left flank pain, nausea, and vomiting and was finally diagnosed with ANHAI.

**Summary**

- Acute non‐hemorrhagic adrenal infarction (ANHAI) should be considered in patients with unexplained abdominal pain, especially those with thrombotic risk factors.
- Early diagnosis via CT imaging and prompt anticoagulation therapy is crucial to prevent complications like adrenal insufficiency, improving patient outcomes.

## 1 Introduction

Acute non‐hemorrhagic adrenal infarction (ANHAI) is a rare and serious condition that results from the obstruction of the adrenal blood supply, typically due to thrombosis of the adrenal vein [1, 2, 3]. Although the adrenal glands are richly vascularized, ischemia can occur due to various thrombotic conditions, including antiphospholipid syndrome, hypercoagulable states, and pregnancy, as well as factors like malignancies, trauma, and surgery [4, 5]. Clinically, patients with ANHAI often present with non‐specific symptoms such as abdominal pain, nausea, or signs of adrenal insufficiency, including electrolyte imbalances [5]. Diagnosis of ANHAI relies on advanced imaging techniques, particularly computed tomography (CT). The rare nature of ANHAI necessitates a high level of attention, particularly in patients with known thrombotic risk factors [6, 7]. Here, we present the case of a middle‐aged female with a history of diabetes mellitus who was diagnosed with ANHAI caused by left renal vein thrombosis. This report underscores the diagnostic challenges and importance of multidisciplinary management in achieving effective patient outcomes for this rare entity.

## 2 Case History/Examination

A 45‐year‐old female with a five‐year history of A 45‐year‐old female with a five‐year history of type 2 diabetes mellitus and chronic obstructive pulmonary disease (COPD) presented to the emergency department with a complaint of nausea and vomiting and severe abdominal pain localized predominantly to the left flank over the past two days. The pain was not radiating, and the patient rated the intensity to be approximately 8/10. It also didn't correlate with specific positions, physical activity, or bowel movements. Furthermore, the patient reported no other local or systemic symptoms, such as fever or urinary tract infection‐related (UTI) complaints, such as dysuria or frequency. She had no history of malignancy. On physical examination, the patient's vital signs were within normal limits. Abdominal examination showed no signs of distension, scar, or herniation. Palpation did not elicit any tenderness, and percussion revealed no evidence of organomegaly. Also, she had diabetic peripheral neuropathy with a socks‐and‐gloves pattern due to her poor control of diabetes. All other findings from the examination were unremarkable.

## 3 Methods (Differential Diagnosis, Investigations, and Treatment)

The electrocardiography disclosed no abnormal finding, and the blood laboratory tests revealed the following: blood glucose level of 350 mg/dL, white blood cell (WBC) count of 17,900/μL with 84% neutrophils, a hemoglobin level of 13.4 g/dL, platelet count of 278,000/μL, prothrombin time (PT) of 13 s, partial thromboplastin time (PTT) of 30 s, and arterial blood biochemistry analysis indicating pH = 7.30, PCO2 = 51 mmHg, and HCO3 = 29 meq/L. Blood electrolyte concentrations were all within normal ranges. However, the dipstick test revealed +2 ketones in the urine. A suspicion was raised for diabetic ketoacidosis (DKA) or hyperosmolar hyperglycemic state (HHS) due to the combination of hyperglycemia and ketonuria. However, because of vomiting (leading to metabolic alkalosis) and underlying COPD (causing respiratory acidosis), the arterial blood gas (ABG) did not reveal the typical metabolic acidosis expected in DKA, and the bicarbonate (HCO₃^−^) level was elevated. As a result, clear metabolic acidosis was absent, and the classic diagnostic criteria for DKA were not fully met. Nevertheless, the patient was in a diabetic crisis and required emergency management similar to that of DKA or HHS.

An initial ultrasound examination showed no remarkable findings. Consequently, non‐contrast‐enhanced and contrast‐enhanced computed tomography (CT) scans (NECT and CECT, respectively) were performed to further investigate the patient's origin of flank pain. Although the NECT revealed no evidence of nephrolithiasis or hydronephrosis, asymmetry and bulging of the left adrenal gland were observed without hyperdense areas indicative of hemorrhage.

**FIGURE 1:**
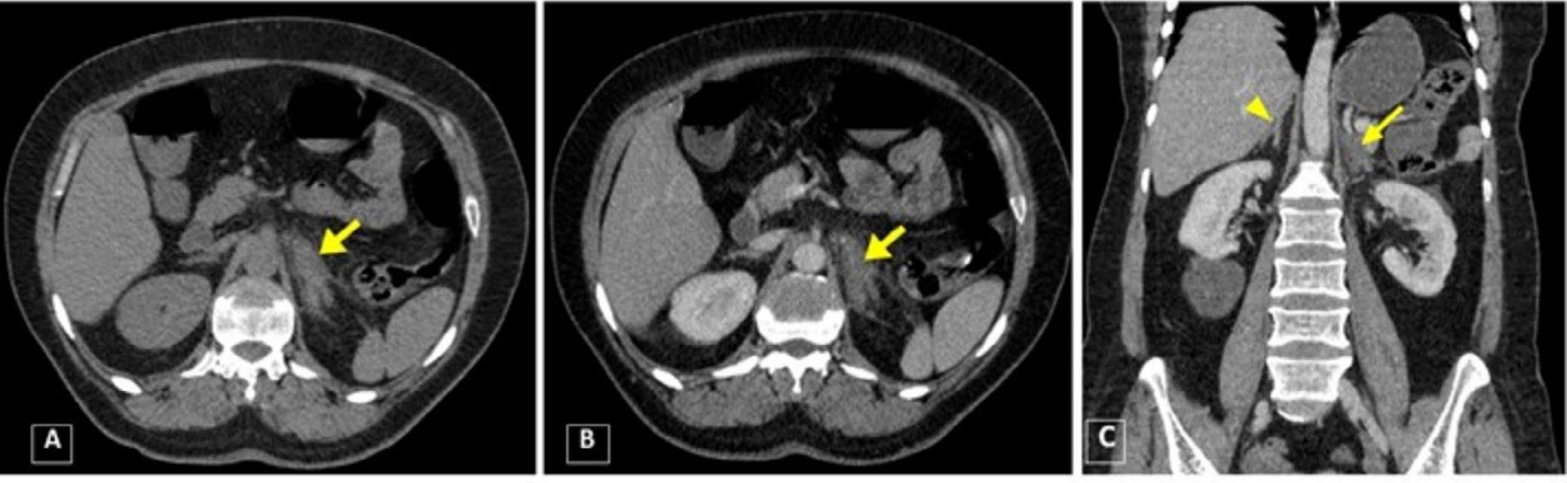
Axial computed tomography: (A) Non‐contrast‐enhanced CT scan (NECT) demonstrated asymmetry and bulging in the left adrenal gland (*arrow*), with no evidence of hyperdensity suggestive of hemorrhage. Furthermore, fat stranding was seen in the left peri‐adrenal space. (B) In the contrast‐enhanced CT scan (CECT), the left adrenal gland (*arrow*) was hypoattenuating with no evidence of enhancement. (C) In the coronal view, the arrow demonstrates that the left adrenal gland is bulged with no enhancement, in contrast to the normal appearance of the right adrenal gland (*arrowhead*). Additionally, a rim of perinephric fluid is noted around the left kidney.

**FIGURE 2:**
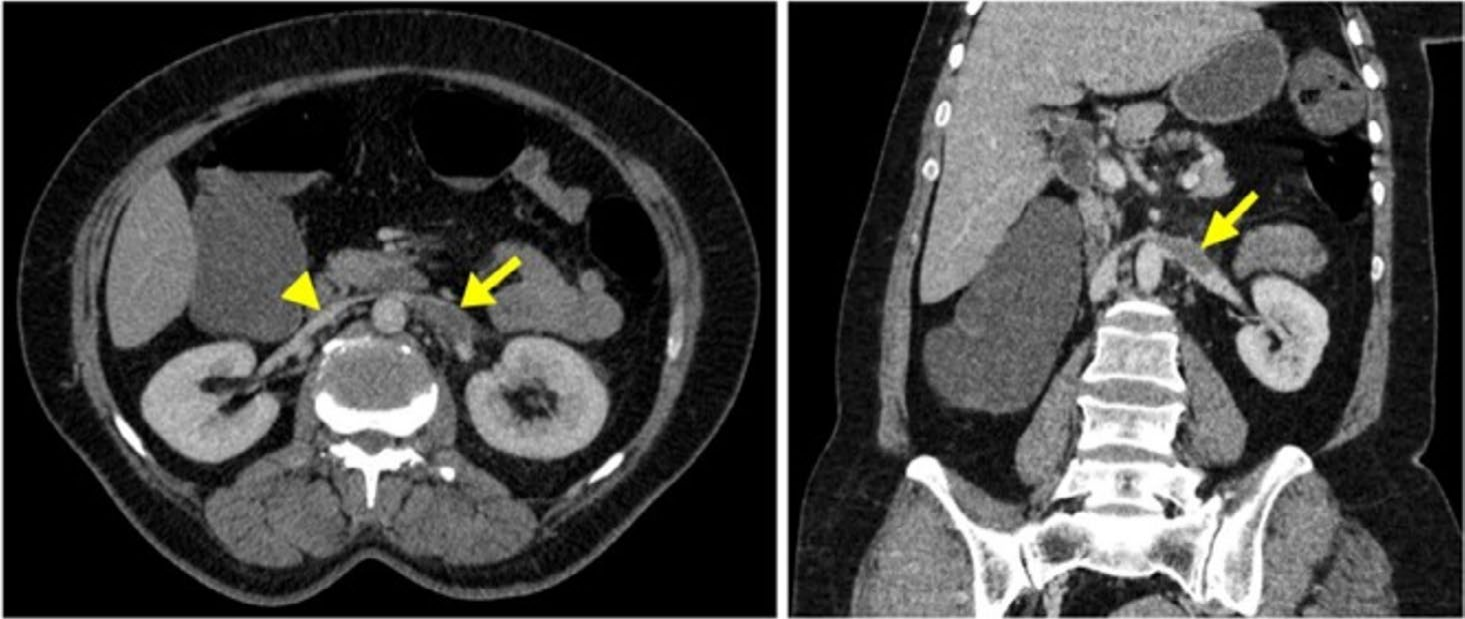
Left: A contrast‐enhanced CT scan (CECT) in the axial plane shows a dilated left renal vein with a hypodense filling defect, which is called thrombosis (*arrow*). Note the right renal vein with normal diameter and completely contrast‐filled (*arrowhead*). Right: In the coronal view, the arrow also indicates left renal vein thrombosis.

The left adrenal gland exhibited no enhancement and appeared hypodense following intravenous contrast administration on CECT. Fat stranding was observed in the periadrenal and perinephric spaces on the left side (Figure 1). Additionally, the left renal vein was dilated and contained a hypodense intraluminal filling defect, indicative of thrombosis, which appeared to extend into the left adrenal vein (Figure 2). There were no remarkable findings in the other parts.

The patient was promptly resuscitated with intravenous fluids, and a diagnosis of ANHAI secondary to left renal vein thrombosis was established. She was immediately started on 5000 IU of low‐molecular‐weight heparin (LMWH). Concurrently, her hyperglycemia was managed with insulin therapy, and her pain was alleviated with narcotics.

## 4 Conclusions and Results (Outcome and Follow‐Up)

To confirm the diagnosis of adrenal infarction, additional laboratory tests were requested. Adrenocorticotropic hormone (ACTH) was 16.0 pg/mL (reference range: 6.0–76.0 pg/mL), cortisol was 15 mcg/dL (5.0–25.0 mcg/dL), and aldosterone was 8 ng/dL (4–51 ng/dL)—all within normal limits.

Additional laboratory tests were conducted to further evaluate potential coagulopathies, which are summarized in Table 1, including hereditary mutations in coagulation‐related genes, complement deficiency, and APS‐related antibodies. These tests were negative in this patient, ruling out any common thrombophilia. Furthermore, blood and urine cultures were reported to be negative, and no source of infection was detected. Following five days of hospitalization, the patient was discharged with a prescription for continued anticoagulant therapy. At the four‐week follow‐up, Doppler ultrasound showed no residual thrombosis in the left renal vein. Additionally, repeat adrenal function tests remained within normal limits, consistent with previous results.

**TABLE 1:** Assessment of additional parameters associated with coagulopathy disorders.

Parameter	Finding	Normal range
Anti‐Nucleus Ab	Negative	Negative
Anti‐DNA Ab	5.5 mg/dL	< 30 mg/dL
Antithrombin III	80%	80%–120%
Anti‐cardiolipin Ab	Negative	Negative
Anti B2glycoprotein I	Negative	Negative
Complement C3	125 mg/dL	90–180 mg/dL
Complement C4	39 mg/dL	10–40 mg/dL
Protein C	94%	55%–120%
Protein S	58%	55%–165%
Factor V Leiden	No mutation	No mutation detected
Homocysteine	3.5 μmol/L	0–12 μmol/L

*Note:* The absence of antiphospholipid antibodies and hereditary thrombophilia markers (e.g., Factor V Leiden, Protein C/S deficiency) is important in ruling out common hypercoagulable conditions that predispose to adrenal infarction.

ANHAI, though rare, should be considered in the differential diagnosis of patients presenting with unexplained abdominal pain, especially those with underlying hypercoagulable conditions or risk factors for thrombosis. Timely recognition and diagnosis are paramount to prevent complications, including adrenal insufficiency, and to ensure appropriate management [5]. Imaging plays a crucial role in diagnosing ANHAI, with CT scans typically showing an enlarged, hypo‐attenuated adrenal gland and the presence of the capsular sign offering valuable diagnostic clues [7]. Early intervention with anticoagulation therapy and supportive measures is essential for managing ANHAI, ultimately improving outcomes for patients [5]. Awareness of this condition, especially in at‐risk populations such as those with a history of thrombosis or pregnant women, is critical for reducing morbidity and preserving adrenal function [1].

## 5 Discussion

Adrenal infarction is a rare yet serious condition that might be life‐threatening if not timely diagnosed [2]. While adrenal infarction often presents as a hemorrhagic event, non‐hemorrhagic cases, known as ANHAI, remain less common and can be even more challenging to diagnose [3]. Adrenal infarction is often hemorrhagic because adrenal glands are richly supplied by the inferior phrenic artery, renal artery, and aorta. Adrenal glands are only susceptible to ischemia when the blood flow to the adrenal veins is entirely obstructed, which is called the ANHAI and is less prevalent compared to hemorrhagic infarction [1]. Hemorrhagic adrenal infarction has the typical appearance of hemorrhage in CT imaging and hence can be distinguished from adrenal neoplasms or abscesses [7, 8].

Literature suggests that 70% of ANHAI cases occur due to adrenal vein obstruction [9]. As a result, any contributing factor or condition related to coagulation disorders could predispose patients to ANHAI. These factors could be hereditary, such as factor V Leiden, Prothrombin G20210A Gene Mutation, Plasminogen Activator Inhibitor‐1 gene mutation, antithrombin, protein C and S deficiency, or acquired, such as pregnancy, antiphospholipid syndrome, malignancy, trauma, major surgery, use of oral contraceptives, hormonal replacement therapy, diabetes, obesity, and smoking [4, 5, 10, 11]. Beyond thrombotic conditions, ANHAI has also been reported in patients experiencing severe hypotension, shock state, and pregnancy [7, 8, 9].

Despite the rarity of ANHAI, pregnancy and antiphospholipid syndrome (APS) have gained notable attention in the literature, as most of the ANHAI cases were reported to be among the population affected by these two conditions [3, 8, 12, 13, 14, 15]. Pregnancy, for example, introduces physiological changes, such as increased blood volume, venous stasis from uterine expansion, and reduced anticoagulant factors, which make pregnant women significantly more predisposed to thrombosis and, consequently, adrenal infarction [3, 12, 16]. Similarly, the main basis for adrenal infarction in APS is vascular events [17]. Based on a theory, adrenal vein thrombosis leads to a hemorrhagic infarction in the adrenal glands [15, 18]. According to another theory, spontaneous non‐thrombotic adrenal hemorrhage may play a role in developing adrenal infarction [15, 18, 19]. However, the exact pathophysiology of adrenal involvement during APS and the clinical course of the disease are still unclear, with limited and inconsistent evidence in the literature.

The challenges in the diagnosis of ANHAI lie in the non‐specific nature of the presenting symptoms and the fact that the adrenal infarction typically does not present with the typical hemorrhagic features, as in the case of other types of infarctions [5]. Adrenal infarction should be considered in the differential diagnosis when patients present with unexplained abdominal or flank pain, particularly if they have known hypercoagulable risk factors. A careful history of potential thrombotic conditions, including recent surgeries, pregnancy, malignancy, or known thrombophilic disorders, should prompt the clinician to consider ANHAI as a possible diagnosis [1]. Furthermore, acute presentation with abdominal pain or signs of adrenal insufficiency (such as hyponatremia, hyperkalemia, or hypoglycemia) should warrant consideration of ANHAI, especially if other causes of abdominal pain are excluded. Adrenal insufficiency can emerge as a consequence of adrenal infarction, leading to clinically significant electrolyte disturbances that could be life‐threatening if left unaddressed [5]. Imaging findings play a crucial role in the diagnosis of ANHAI. The lack of enhancement on contrast‐enhanced computed tomography (CT), as well as the characteristic hypoenhancing appearance of the adrenal gland, is key to distinguishing ANHAI from other causes of abdominal pain, including potential malignancy or abscess formation. The “capsular sign,” defined by a subtle line of peripheral hyperenhancement, can further help identify this rare condition on imaging [6, 7].

Prompt recognition of this rare disorder is essential, as it could potentially prevent more severe sequelae, including complete adrenal failure [15]. When diagnosed early, treatment strategies typically include supportive therapy with volume resuscitation and the use of anticoagulants to prevent further thrombosis, particularly in patients with underlying coagulopathies. Although initial therapies can lead to improved clinical outcomes, ongoing surveillance with imaging is essential to ensure the resolution of thrombotic events and recovery of adrenal function [5, 20].

In this case, we presented a middle‐aged diabetic woman who presented with a complaint of non‐tender, severe left flank pain, nausea, and vomiting for two prior days. The patient's vital signs were within normal ranges, showing no signs of clinical deterioration. The laboratory investigations revealed hyperglycemia, neutrophil‐dominant leukocytosis, and mixed metabolic‐respiratory acidosis despite no signs of electrolyte imbalance or any abnormalities in coagulation test results. On further investigation, the left adrenal appearance was compatible with the pattern of ANHAI on the CT scan secondary to left adrenal vein thrombosis. The abdominal pain in this patient could easily be attributed to diabetic ketoacidosis (DKA) and led to missing the diagnosis of ANHAI. However, due to the localization of pain in the left flank region, the physicians suspected other causes rather than DKA and performed an initial sonography examination.

Previous case studies aimed to report the origin of ANHAI. Recently, Tschuertz et al. highlighted pregnancy as a leading cause of ANHAI and hence conducted a review study to summarize the findings of 19 papers. Out of 24 ANHAI cases found to be due to pregnancy, they reported that severe non‐relieving abdominal pain, mostly accompanied by nausea and vomiting, was the most prominent sign during the investigations, which required opioid analgesics in most cases. The MRI and CT scans contributed to the confirmation of diagnosis approximately equally, and in a significant fraction of cases, multimodal imaging was leveraged using both CT and MRI scans. Furthermore, ANHAI was recorded two times higher on the right adrenal gland. Anticoagulant therapy with heparin was initiated for three‐fourths of cases; however, major hypercoagulopathy disorders were only detected in one‐third of cases following further investigations. Laboratory findings were also non‐diagnostic in most of the cases. Adrenal insufficiency was a rare complication of ANHAI in these cases, being recorded in only one patient [12]. Except for the side of ANHAI, this description mostly aligns with the presentation of our patient; however, she wasn't recorded or revealed to be pregnant.

Liang et al. also reported a case of ANHAI following simultaneous systemic lupus erythematosus (SLE) and APS. The patient's clinical presentation involved pain in the fossa axillaries and the inguinal region. The diagnosis was made after the CT scan confirmed the appearance of ANHAI in bilateral adrenal glands and the laboratory findings revealed a decrease in C3 and C4 complements, as well as the positivity of auto‐immune antibodies related to SLE and APS (ANA, Anti‐dsDNA antibody, Anti‐Smith antibody, Anti‐SS‐A antibody, Anti‐SS‐B antibody, …) [13]. However, the laboratory investigation in our case didn't result in the detection of auto‐immune antibodies related to APS or SLE; moreover, the CT scan showed thrombosis in the left adrenal vein. Although the patient was fully treated with anticoagulation, insulin, and fluid therapy, additional work‐up to address the underlying hypercoagulable state yielded no significant findings, as the findings were negative for the APS and the most common hereditary coagulation disorders. Following four weeks, the thrombosis was fully resolved on the follow‐up Doppler ultrasonography. Moreover, although the patient showed no clinical or laboratory signs of adrenal insufficiency, normal cortisol and ACTH levels were confirmed to rule out subclinical adrenal dysfunction. Including adrenal function assessment remains essential in similar cases, given the risk of delayed or evolving insufficiency.

In summary, although the exact cause of thrombosis in the left adrenal vein remained undetermined in our patient, early diagnosis and prompt anticoagulant therapy led to the complete resolution of thrombosis within four weeks. We hypothesize that diabetic ketoacidosis may have contributed to venous thrombosis and subsequent ANHAI; however, this remains speculative and warrants further investigation in future studies, as, to the best of our knowledge, no existing literature has established a direct link between diabetic ketoacidosis and ANHAI.

Long‐term follow‐up in patients with ANHAI is essential to monitor for potential recurrence of thrombotic events and delayed adrenal insufficiency. Regular adrenal function testing and imaging may be warranted in select cases, especially when initial infarction is extensive or bilateral involvement is suspected.

## Author Contributions

**Parvaneh Layegh:** resources, supervision, writing – review and editing. **Atiyeh Mahdavi Rafie:** project administration, writing – original draft, writing – review and editing. **Armin Doostparast:** writing – original draft, writing – review and editing.

## Consent

Written informed consent was obtained from the patient for publication of this report in accordance with the journal's patient consent policy.

## Conflicts of Interest

The authors declare no conflicts of interest.

## Data Availability

All data generated or analyzed during this study is included in the manuscript.
